# The Biology of the Nuclear Envelope and Its Implications in Cancer Biology

**DOI:** 10.3390/ijms20102586

**Published:** 2019-05-27

**Authors:** Maria Alvarado-Kristensson, Catalina Ana Rosselló

**Affiliations:** 1 Molecular Pathology, Department of Translational Medicine, Lund University, Skåne University Hospital, 20502 Malmö, Sweden; maria.alvarado-kristensson@med.lu.se; 2 Laboratory of Molecular Cell Biomedicine, University of the Balearic Islands, 07121 Palma de Mallorca, Spain; 3 Lipopharma Therapeutics, Isaac Newton, 07121 Palma de Mallorca, Spain

**Keywords:** nucleus, envelope, γ-tubulin, mitosis, cell cycle, signaling, genome, cancer, diagnosis, metastasis

## Abstract

The formation of the nuclear envelope and the subsequent compartmentalization of the genome is a defining feature of eukaryotes. Traditionally, the nuclear envelope was purely viewed as a physical barrier to preserve genetic material in eukaryotic cells. However, in the last few decades, it has been revealed to be a critical cellular component in controlling gene expression and has been implicated in several human diseases. In cancer, the relevance of the cell nucleus was first reported in the mid-1800s when an altered nuclear morphology was observed in tumor cells. This review aims to give a current and comprehensive view of the role of the nuclear envelope on cancer first by recapitulating the changes of the nuclear envelope during cell division, second, by reviewing the role of the nuclear envelope in cell cycle regulation, signaling, and the regulation of the genome, and finally, by addressing the nuclear envelope link to cell migration and metastasis and its use in cancer prognosis.

## 1. Introduction

Papanicolaou’s smear test [1] is a routine check for uterine and cervical cancer. In addition, it has been implemented for a variety of specimens, such as fine needle aspiration biopsies, cerebrospinal fluid, pleural fluid, and urine samples, among others [2,3]. This cancer diagnosis test is based on a combination of changes in the staining, size, and shape of the nuclear chromatin. The eukaryotic nucleus encloses and regulates this chromatin by using a double membrane (nuclear envelope, NE), which, in turn, provides a unique molecular and biochemical environmental protective mechanism against potentially damaging cytoplasmic enzymatic activities, such as oxidative metabolism.

NE includes three interconnected domains with morphological differences: The inner nuclear membrane (INM) and outer nuclear membrane (ONM) and the pore membranes. These two individual lipid bilayers are separated by a luminal space of 30–50 nm in human cells, named the lumen or perinuclear space. For the transport of macromolecules in and out of the nucleus, both NM connect to form pore membranes where the nuclear pore complexes (NPCs) responsible for transport regulation are inserted [4,5,6]. Indeed, INM and ONM form discrete domains of a single membrane system separated by the NPCs [4,5]. The ONM is contiguous with the rough endoplasmic reticulum (ER) and contains ribosomes on its outer surface [4,5]. Furthermore, the ONM is connected to the cytoskeleton through its integral proteins, and in turn, these proteins connect to the luminal parts of INM proteins at the luminal space, all in all connecting the cytoskeleton to the nucleoskeleton and chromatin. Although INM, ONM, pore membranes, and ER originate from a continuous structure, they maintain their identities to a large extent through unique profiles of integral-membrane and other associated proteins, together with specific compositions of lipids and cholesterol [7,8,9]. In addition, the protein composition of the nuclear envelope membranes is variable across different mammalian tissues (reviewed in Reference [10]).

In metazoan organisms, the primary scaffold of NE is provided by a fibrous layer called the nuclear lamina. This thick protein layer that underlies the INM has been described in studies with isolated NEs, since nuclear lamina is resistant to most chemical extractions used in biology. The most abundant proteins of the nuclear lamina are, by far, three polypeptides of around 65–70 kDa that correspond to type V intermediate filament proteins and are named lamins [11,12]. It is estimated that there are roughly 3,000,000 copies of lamins in a typical mammalian nucleus [13]. The specific lamin nucleoskeleton is distinct from the nuclear matrix that supports chromatin inside the nucleus. Four lamin proteins are expressed in mammalian somatic cells. A-type lamins (lamins A and C) are produced by alternative mRNA splicing of *LMNA* gene, whereas B-type lamins (lamin B1 and B2) are encoded, respectively, by *LMNB1* and *LMNB2* genes. Lamins C2 and B3 are germ-cell-specific isoforms produced by alternative splicing of *LMNA* and *LMNB1*, respectively. B-type lamin expression occurs early in embryonic development and persists ubiquitously through adult life. In contrast, the A-type lamins are expressed in an asynchronous and developmentally regulated manner and are only detected after tissue differentiation (reviewed in [14]). Indeed, before day 10 of murine embryonic development, no A-type lamins are detected in the embryo proper [15,16]. The last constituent of the nuclear lamina is a collection of integral and associated proteins of the INM.

The gatekeeper function necessary for the translocation of proteins in and out of the nucleus is controlled principally by the NPC. The NPCs are large assemblies of >60 MDa in mammals. Several copies of a set of 30 diverse proteins, termed nucleoporins (NUPs), are found in these structures (reviewed in [6]). Only a restricted number of structural domains are found in the nucleoporins sequence. Those domains are transmembrane domains, Phe-Gly (FG) repeats, WD domains, α-helices, and β-propellers [17,18,19]. Around 2000–3000 NPC units are found in an average mammalian nucleus. FG-NUPs, in particular, play the most relevant role in defining the NPC diffusion limit and the list of nuclear transport receptors that might be shuttled through the NPC ([20] and reviewed in Ref. [21]). Indeed, NPCs are not a mere gateway to the nucleus. First, the size and maturation of nuclear pores is a crucial event to the nuclear import and nuclear growth and size at the end of mitosis [22,23], reviewed in Ref. [24] and during interphase [23,25,26], as shown in several vertebrate models. Second, NPCs might alter nuclear morphology through the physical link with lamins [27]. Third, nucleoporins are a model of long-term protein endurance: NPCs are sustained over the lifetime of a cell by means of a slow but finite interchange of its steadily more stable subcomplexes [28]. In tumorigenesis, the incidental finding of Nup88 as a biomarker of cancer [29,30] opened the door to detection of high levels of Nup88 in several types of tumors [30,31,32,33,34,35,36]. Beyond this protein, only a small number of nucleoporins have been associated with tumorigenesis. These include proteins Nup62, Nup88, Nup98, Nup214, and Nup358/RanBP2, all of them elements of the trafficking pathway and are specifically related to the export of mRNA (reviewed in Ref. [37]). In the literature, increases in the number of NPCs were related with more aggressive tumors [38,39,40,41].

In addition, nuclear envelope transmembrane (NET) proteins are also found both INM and ONM. This host of integral proteins might be tissue-specific for up to 60% of their protein elements [42]. Most NETs bind lamins and interact with chromatin (reviewed in [43]). The most known NET is emerin, which is encoded by the *EMD* gene in humans. Emerin is a founding member of the LEM domain-containing integral proteins of the inner nuclear membrane in vertebrates, where LEM is named for LAP2, emerin, and MAN1 (reviewed in [44]). Emerin is highly expressed in cardiac and skeletal muscle and several mutations affecting this gene cause X-linked recessive Emery–Dreifuss muscular dystrophy (EDMD).

More than preserving genetic material and providing architecture and mechanical support in eukaryotic cells, the NE is a key cellular hub that plays a dynamic role in the control of cell cycle regulation, mitosis, apoptosis, DNA repair, ageing, nuclear architecture, signaling, chromatin organization, gene expression regulation, and cell migration [45,46,47,48,49,50,51]. All of these various functions are critical for the processes of tumorigenesis, tumor growth, and metastasis. All in all, it is reasonable that the diagnosis of cancer relies on morphologically distinctive alterations in the NE that are only recognizable by the eye of a well-trained pathologist.

## 2. The Nuclear Envelope in Cell Division

Cancer is the result of uncontrolled cell division. NE proteins can mostly affect the cell cycle in higher eukaryotes when the cells undergo open mitosis and the nucleus architecture is dismantled to allow the partitioning of the genetic material between the daughter cells. Indeed, the finding in mammalian cells of the depolymerization of lamin polymers upon hyperphosphorylation of lamin A at the onset of mitosis was the first clue in NE regulating cell division [52,53,54,55].

### 2.1. Nuclear Envelope Disassembly at the Onset of Cell Division

Phosphorylation of NE proteins and of their binding partners drives the coordinated disruption of NE interactions and structures at the beginning of mitosis. Together with lamins, several NPC proteins and NETs are also phosphorylated by mitotic kinases (gp210, LAP2β, and lamin B receptor –LBR), as shown in human, murine, or avian models [56,57,58,59]. In human and *Caenorhabditis elegans* cells, the same occurs with barrier-to-autointegration-factor (BAF), a chromatin binding partner of several NETs connecting chromatin to NE [60,61] (reviewed in [62]).

Disassembly of the NE needs close coordination with the generation of the bipolar mitotic spindle. In prophase, NPC-attached dynein motors assist in the separation of the centrosomes [63,64].

Disassembly of NPCs is not a straight reversal of the assembly steps (reviewed in [65,66]). In many cases, components of the NE and NPCs actively participate in mitotic events when released from their interphase organization [67]. At the G2/M cell cycle transition, two nucleoporins participate in tethering centrosomes to the NE [68,69]. During prophase, these interactions might help microtubules in their function for NE breakdown [70] and for moving of sister centrosomes to opposite sides of the nucleus [68,69,71]. At the end of prophase, the NPC is dismantled releasing elements with important regulatory functions during mitosis: NUP358 at kinetochore functioning [72,73,74], NUP88 and other nucleoporins interfering with microtubule dynamics to promote spindle assembly, NUP98 in regulating the adenomatous polyposis coli (APC)/C [67,75,76,77] (reviewed in [65]).

During mitosis in animal cells, remodeled nuclear membranes intermix in a large part with the tubulo-vesicular mitotic ER [78], while NE vesiculation also occurs [79,80,81,82,83,84,85,86]. Regarding the over 100 different NETs in any given cell, several of them go into a storage form and others exert critical functions, such as RanGTP, the transport receptor importin/karyopherinβ, and RepoMan ([87], reviewed in Ref. [6,88,89]).

Several features of cancer cells, such as lagging chromosomes, aneuploidy, and polyploidy, might occur after a failed NE breakdown at the onset of mitosis and the subsequent blocking of spindle assembly.

### 2.2. Nuclear Assembly After Cell Division

#### 2.2.1. Chromatin Enclosing, INM Protein Recruitment, and NPC Formation

During metazoan anaphase, chromosomes cluster compactly together in a disc-like configuration whose surface drives nuclear assembly. During early telophase, NE reassembly is initiated by changes at this chromatin surface (i.e., removal of mitotic histone marks by phosphatases) and the dephosphorylation-induced binding of NETs and their associated membranes to chromatin (reviewed in [78]).

**Figure 1:**
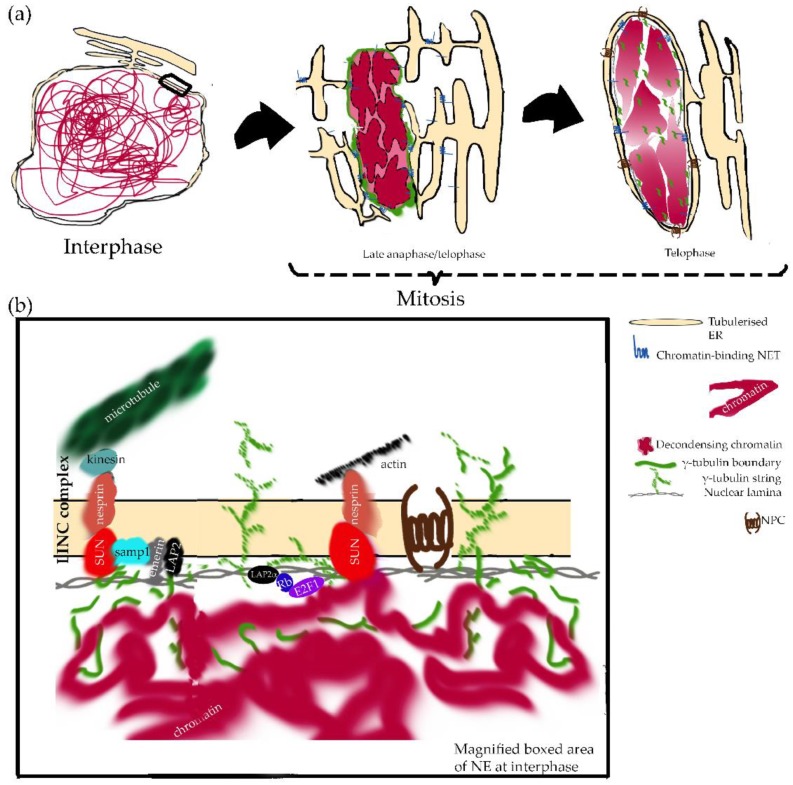
A model for the role of nuclear γ-tubulin in chromatin enclosing, inner nuclear membrane (INM) protein recruitment, and nuclear pore complex (NPC) formation in nuclear disassembly and reassembly upon cell division. (**a**) Schematic representation of the nucleus and nuclear envelope (NE) during interphase and subsequent mitosis. Post-mitotic reassembly of the NE is initiated by changes at the chromatin surface (i.e., removal of mitotic histone marks by phosphatases) and dephosphorylation-induced binding of nuclear envelope transmembrane proteins (NETs) and their associated membranes to chromatin. Engulfment of recently separated sister chromatids by the NE occurs via deposits of membrane fragments on the chromatin surface that trigger the enveloping process. The NE is reformed from endoplasmic reticulum (ER) membranes, which contact chromatin either as tubules or sheets. After membrane deposition, NPC assembly allows importation of the elements for nuclear lamina assembly. Microtubule organizer γ-tubulin plays a distinct role in promoting this NE formation by providing a γ-tubulin boundary indispensable for NE assembly. At the onset of mitosis, the lamina meshwork is disrupted, but the γ-tubulin boundary around the mitotic chromosomes is maintained. During mitosis, chromatin-associated γ-strings link the sister chromatids to the cytosolic γ-string pool. Finally, at anaphase/telophase, the γ-tubulin boundary composed of cytosolic and chromatin-associated γ-strings forms a supporting scaffold that assists the formation of the nuclear envelope. (**b**) Magnified boxed area of NE at interphase: During interphase, γ-tubulin bridges connect the cytosolic and the nuclear γ-tubulin pools ([123], reviewed in [128,129]). The LINC protein nesprins recruit centrosomal proteins and regulate the nucleation of microtubules from the NE in myotubes [130]. The INM protein Samp1 is in contact with both γ-tubulin and SUN1 [131]. At the beginning of mitosis, during the rupture of the NE to the spindle microtubules, Samp1 might recruit γ-tubulin from the fenestrated NE. In the spindle, γ-tubulin and Samp1 complex together with augmins would potentially assist in the nucleation of the microtubules. Not to scale.

Several mechanisms combine to recruit ONM and INM proteins, constituents of NPCs, and lamins. In metazoa, INM proteins are attracted both by both specific interactions and by the general affinity of many INM proteins for chromatin/DNA, for example, LBR binds heterochromatin protein 1 (HP1) [90,91] and histone H3 [92,93,94]. Importantly, NET and NPC binding to mitotic chromosomes in early telophase seems to drive NE reassembly [95,96,97,98,99], implicating phenylalanine and glycine (FG)-rich nucleoporins and the AT-hook-domain containing protein, ELYS/Mel-28 (Figure 1). ELYS localizes not only to NPCs, but is also associated with chromosomal kinetochores during cell division. At the end of mitosis, ELYS recruits the NUP107-160 subcomplex, which is required for the correct segregation of mitotic chromosomes [100,101]. In addition, in NPC assembly and chromatin decondensation, lysine demethylase LSD1 is required [102], while the Repo-Man-promoted dephosphorylation of histone H3 seems indispensable for targeting importin-b to mitotic chromatin [103] (Figure 1).

Engulfment of recently separated sister chromatids by the NE occurs in an astonishingly short timeframe thanks to deposits of membrane fragments on the chromatin surface that trigger the enveloping process (reviewed in [78,104]). It is still a matter of debate whether ER moves toward chromatin in the way of membrane sheets or tubules (reviewed in [105]). Regardless of the case, defects in any of these mitotic functions could affect the quality of cell division and lead to aneuploidy, a common feature of tumors [106]. In the metazoa, NPC formation occurs in two different phases of the cell cycle and through different assembly mechanisms: Post-mitotic NPC assembly and interphase NPC formation [18,107,108,109,110]. Two models for post-mitotic NPC assembly have been proposed: The insertion model claims that NPCs are reassembled into an intact nuclear envelope, while the enclosure model proposes that NPC assembly starts before the NE encloses the chromatin (reviewed in [111,112,113]). In any case, post-mitotic NPC assembly happens in a step-wise manner and it is subjected to fine surveillance mechanisms (reviewed in [65]). NPC assembly begins in early anaphase with soluble NPC proteins (Nup107-160 scaffold) positioning on the chromatin, mediated by Elys/Mel28, before membrane reformation. Then, it might be followed by the recruitment of transmembrane nucleoporins (reviewed in [24,108,109]). In the case of NUP153, it might even participate in the biogenesis of the lamina [114].

Surveillance mechanisms ensure correct post-mitotic reformation of NPCs (reviewed in [65]) and the assembly of the basket-like feature is particularly necessary to complete cytokinesis in a timely manner [114]. Aurora kinase B links the basket-like feature with cytokinesis, and this is currently explored as a chemotherapeutic approach in clinical trials against cancer [115].

The number of NPCs formed during interphase doubles prior re-entry into mitosis (reviewed in [111]). However, little is known about NPC formation during interphase, although it is differentially regulated compared to post-mitotic NPC assembly. Interphase NPC formation is dependent upon cyclin-dependent kinase (CDK) activity, but not upon ELYS/Mel-28 [116,117].

Kinetochores and microtubules are also essential in NE reassembly, particularly, in the recruitment of BAF to the chromatin template [118]. Acetylated Lem4 (ANKLE2) participates in this process by promoting the dephosphorylation of BAF [96,97,119]. The binding of BAF to chromatin is indispensable for most of the integral-membrane LEM-domain containing proteins to connect to chromatin through their interaction with BAF itself [118,120,121]. A recent elegant report using human cells showed that the role of BAF in nuclear assembly depends upon its ability to link distant DNA sites [122]. Microtubule organizer γ-tubulin may play a noncanonical and distinct role in promoting NE assembly [123]. A local suppression of microtubules during nuclear formation, fulfilled by chromatin-bound microtubule regulators, is required in *X. laevis* for proper pronuclear assembly and regular morphology of the nucleus [124]. An association of γ-tubulin with the nucleoporin ELYS/Mel-28 and the NE reassembling GTPase, Ran, has been described in *X. laevis* [125]. In both *X. laevis* and mammalian cells, a γ-tubulin boundary made of γ-strings is formed around chromatin during NE assembly, and this γ-tubulin boundary ensures the formation of the lamina around chromatin by recruiting of lamin B (Figure 1) [123]. The formation of fibrillar aggregates of γ-tubulin was further confirmed upon chaperonin containing TCP-1 (CCT) action in vitro [126]. Shaping the nucleus and achieving a regular distribution of NPCs has been shown to depend upon the γ-tubulin complex protein 3-interacting proteins in *Arabidopsis thaliana* [127]. Furthermore, in human cells, expression of a γ-tubulin mutant that lacks the DNA-binding domain forms chromatin-empty nuclear-like structures, demonstrating that a persistent interplay between the chromatin-associated and the cytosolic pools of γ-tubulin is required for proper NE assembly [123].

The finding that several NETs (NET5/Samp1/Tmem201, WFS1, Tmem214, and otefin) partially concentrate on or around the mitotic spindle, and in the case of the latter, the centrosome during mitosis together with the tissue specificity of many of the NETs affecting the cell cycle, suggests that further implications of these proteins in mitosis might come in the future (reviewed in [14]).

#### 2.2.2. Spatial Distribution of NE Elements

Chromatin discs involve different areas: the “inner core” (the central region of the disc that faces the midzone), the “outer core” (the central region that faces away), and the “non-core” region (the peripheral edge of the disc) [16,118,132,133].

In metazoa, INM proteins and membrane destined for the NE make initial contacts at the non-core region with the γ-tubulin boundary before spreading around and engulfing chromatin [123,134]. Telomeric regions of sister chromatids are bound with unique proteins essential to nuclear architecture, as is LAP2α [98,99]. Furthermore, in newly forming nuclei, telomeres localize to the periphery of the nucleus, suggesting that these regions are involved in the initial seed of nuclear assembly [135].

The core region (inner and outer) is the target for ESCRT (endosomal sorting complexes required for transport) pathway proteins to recruit the microtubule severing factor spastin and seal annular gaps in the newly formed NE [136,137,138,139]. This core is initially deficient in NPC formation, but the process begins at this site soon after membrane closure [133].

The relevance of NPCs in anchoring interactions necessary for nuclear shape maintenance and structural integrity is illustrated, first, by interactions between nucleoporins NUP53, NUP88 and NUP153 and lamins [140,141,142], second, by the finding of polymorphic, lobular nuclear shapes after the depletions of these nucleoporins [140,143,144], and third, by SUN domain-containing protein 1 (SUN1) preferential location in the vicinity of NPCs [145].

All in all, defects in NE proteins might cause an inability to disassemble the NE at mitosis onset (generating partially maintained connections between NE fragments and chromatin) and to reassemble NE at the end of mitosis, blocking proper chromosome segregation and resulting in micronuclei and aneuploidy [106]. The wrapping of all chromosomes into a sole nucleus is thus essential for preserving the integrity of the genome and preventing the development of tumors.

## 3. The Nuclear Envelope in Cell Cycle Regulation and Signaling

### 3.1. Nuclear Envelope in Cell Cycle Regulation

Several elements of the NE (lamin A, lamin B, LAP2α, γ-tubulin, and emerin) have been shown to interfere with the function of the main effectors of cell cycle regulation (retinoblastoma protein–RB, E2Fs, c-Myc), as reviewed below.

In the mammalian cell cycle, normal cells exert a tight regulation of the G1-to-S phase transition, whereas in cancer cells, this transition is a main objective for dysregulation. RB is one of the earlier identified tumor suppressors [146]. Hence, RB activity is deregulated in a broad spectrum of tumors [147]. RB has abundant binding partners [148], the most important of which is the transcriptional factor E2F, which controls a range of genes important for entry into the S phase of the cell cycle. Hypophosphorylated RB binds to E2F complexes and represses the expression of S-phase genes, retaining cells in G1. CDK-dependent phosphorylation promotes the release of RB from E2F and cell cycle progression [149].

In mammals, lamin A regulates G1-to-S phase transition by affecting the RB pathway [150,151,152,153,154], since A-type lamins are required for proper RB function. In detail, A-type lamins promote RB-dependent transcriptional repression of E2F target genes. Furthermore, A-type lamins influence three other machineries regulating RB function: RB phosphorylation, RB localization, and RB protein stability [155,156]. The effect of A-type lamins in RB protein stability, together with the altered activity of ubiquitin ligase components detected in cells expressing mutant forms of lamin A, raise the possibility that A-type lamins work as coordinators of nuclear proteasome function [157].

The RB pathway is further implicated in telomere regulation and cell senescence and cell differentiation in multiple lineage, DNA replication, mitosis, and DNA-damage-activated checkpoint pathways (among others) [147], further linking A-type lamins to all of these processes. Supporting the implication of lamins in the regulation of DNA replication, intranuclear A-type lamins have been shown to associate with initial sites of DNA synthesis upon S-phase entry [158]. In immortalized cells, lamin B was localized to intranuclear sites of late S-phase replication [159], and disruption of the lamin structure impairs initiation of DNA synthesis [160,161,162].

More than A-type lamins, nuclear γ-tubulin also regulate the transcriptional activity of E2F [163]. Nuclear γ-tubulin and E2F concur in a DNA-binding complex isolated from E2F-regulated promoters [163]. In addition, RB1 and γ-tubulin proteins mutually control their expression, and, in several tumors, an inverse correlation in their expression levels was reported for γ-tubulin and RB1 [164]. Interestingly, γ-tubulin also interacts with lamin B recruitment at post-mitotic NE reassembly, as previously mentioned [123].

Other A-type lamin functions may promote G1 maintenance, since RB–lamin A/C and extracellular signal-regulated kinase (ERK)1/2–lamin A/C complexes are mutually exclusive. When G1 arrested cells are stimulated with serum, c-Fos protein is phosphorylated by mitogen activated protein kinase (MAPK) ERK1/2. Phosphorylated c-Fos associates with c-Jun- to form a dimeric Activating Protein 1 (AP-1) transcription activator complex that mediates cell cycle progression [165]. ERK1/2-dependent lamin A/C binding upon serum stimulation releases RB from the RB–lamin A/C complex, thereby promoting cell cycle progression.

The NPC-associated sentrin-specific protease 1 (SENP1) is also reported to influence cell cycle progression by regulating the expression of CDK inhibitors [166,167].

Regarding *c-Myc*-encoded proteins, their association with the nuclear matrix was first described in avian nuclei [168], and more recently, it was shown that the stabilized and active form of the MYC protein (pS62MYC) is enriched at the nuclear periphery of mammalian cell lines in proximity with lamin A/C [169], and precisely localizes to the nuclear pore basket [170]. However, how this regulates transcription and cellular functions remains to be elucidated.

Concerning cell senescence, the NE and the RB pathway have been implicated in an oncogenic signaling that triggers a cell cycle arrest program, i.e., oncogene-induced senescence (OIS). A dramatic reorganization of heterochromatin occurs in OIS. OIS cells lose heterochromatin interactions with lamin B1 through lamina-associated domains (LADs) [171,172], therefore, heterochromatin leaves the nuclear periphery and appears as internal senescence-associated heterochromatin foci (SAHFs) [173]. The activation of the pRB pathway is implicated in the appearance of SAHFs [173], while the NE is also implicated via laminB1 and LBR [174,175] and nuclear pore density [176]. In addition, the composition and density of the NPC changes during differentiation and tumorigenesis [19,46,176,177,178].

With respect to the maintenance of telomere metabolism [179] and DNA damage, in human cells, mutant LMNA has been connected to p53 engagement due to enhanced DNA damage (reviewed in [180]). Indeed, retinoblastoma independent regulation of cell proliferation and senescence by the p53-p21 axis was reported in lamin A/C-depleted cells [181].

In apoptosis, both via the intrinsic and extrinsic pathways, lamins have been described as cleaved by caspases 3 and 6. Indeed, the cleavage of lamin proteins by caspases is a necessary step in apoptosis that allows for nuclear membrane degradation to proceed, followed by chromatin condensation in a murine model [182]. In human and avian cells, A-type lamins are cleaved at their conserved VEID site by caspase 6, while B-type are cleaved at their VEVD site by caspase 3 [183,184,185]. In contrast, an active role of lamins in the induction, but also the prevention of apoptosis is beginning to emerge (reviewed in [186]). In cancer, apoptosis is usually reported. Strikingly, apoptosis levels are increased in the most highly proliferative tumors compared to lowly proliferative tumors. The role of lamins, if any, behind these altered levels is still unclear. One possibility would be that the amount of lamins present and the accessibility of lamins for caspases could delay the onset of apoptosis in certain tumors [187].

In metazoan, an estimated 10% of total A-type lamins localize throughout the nucleoplasm in a mobile and dynamic pool, most likely in association with LAP2α [98,162,188]. Studies on the role of A-type lamins and the RB pathway do not discriminate between these two lamin pools. However, the LAP2α promoter was reported to bind E2F1 and c-Myc [65], E2F1 and E2F4 [66], E2F3b [67], and E2F7 [68], as assessed by chromatin immunoprecipitation and microarray techniques. Indeed, LAP2α expression aligns with the phase of the cell cycle, and its overexpression has been described in various human tumor samples and cancer-derived cell lines (reviewed in [189]).

A last example is the INM protein emerin, which has been linked to cell cycle misregulation in microarray studies in X-linked EDMD patient samples where the lack of emerin disrupts the RB pathway [190].

In summary, several elements of the NE interact with the regulators of cell cycle progression, cell senescence, telomer metabolism and apoptosis. Therefore, perturbations in NE elements affecting the strict control of these interactions can lead to the development of cancer.

### 3.2. Nuclear Envelope in Cell Signaling

Extracellular or cytoplasmic stimuli reach the nuclear interior through signal transduction with the aim of inducing a cellular response, resolved mainly through variations in gene expression. The following signaling cascades from the plasma membrane count with an additional layer of regulation at the NE: MAPK signaling, AKT-Mammalian Target of Rapamycin signaling, Notch signaling, Wnt signaling, NF-κB signaling, and transforming growth factor-β (TGFβ) signaling. This control comes from the fact that signaling cascades need to get into the nucleus through the NPCs and that several effectors (β-catenin and smads) are sequestered at the NE by multiple NETs, as described below.

#### 3.2.1. Lamins in Cell Signaling

The MAPK pathway dysregulation has been shown to be a driving factor in oncogenesis [191,192,193]. This pathway involves three main arms: ERK1/2, c-Jun NH_2_-terminal kinase (JNK), and p38 [194,195]. Phosphorylated MAPKs transit to specific subcellular compartments, such as the nucleus, to elicit their function. The localization of phosphorylated MAPKs to the nucleus is predominantly mediated by binding interactions with sequestering anchors and components of the nuclear transport machinery. In the nucleus, phosphorylated MAPKs regulate various cellular processes from growth to apoptosis, passing through differentiation, inflammation, metabolism, stress response, and autophagy [194,195,196]. An ERK1/2-activated transcription factor promoting cell cycle progression is, as previously introduced, c-Fos. c-Fos activity is suppressed by a sequestering interaction with lamin A that localizes this transcription factor to the NE [197]. Furthermore, ERK1/2 colocalizes with c-Fos at the NE by means of binding lamin A and this leads to the phosphorylation and release of c-Fos from the NE in mammalian models [198]. In addition, enhanced nuclear accumulation of ERK1/2 and JNK was reported in mice carrying a missense mutation that causes autosomal dominant EDMD in humans [199].

The AKT- mammalian target of rapamycin (mTOR) signaling pathway is frequently co-activated along with ERK1/2 in response to growth factor signaling and in various forms of cancer [200,201]. Alterations in A-type lamins have been shown to trigger AKT-mTOR signaling in the above-mentioned mice model of autosomal dominant EDMD and in mice expressing a truncated form of lamin A (lamin AΔ8-11). In mammals, lamin A itself can be phosphorylated by AKT, by which its expression can be regulated [202,203].

Hutchinson–Gilford progeria syndrome (HGPS) is mainly linked to a silent mutation *LMNA*. This G608G mutation in *LMNA* triggers a cryptic splice site and as a result, the progerin protein is produced. Progerin is a truncated form of prelamin A where the last C-terminal 50 amino acids are missing [204,205]. Several signaling pathways are imbalanced in HPGS due to the presence of progerin:

- One of them is Notch signaling, which is altered in many cancers and is thought to maintain cancer stem cells [206]. This highly conserved juxtacrine signaling is involved in regulating cell fate specification and it is altered in the mesenchymal stem cell lineage in HPGS.
- Additionally, the deposition of extracellular matrix (ECM) is altered in children with HGPS, mainly due to the reduced activity of the TCF4/LEF1 complex, a key downstream effector of the Wnt signaling pathway. Progerin expression decreases the expression and nuclear accumulation of LEF1 [207].
- NF-κB signaling functions as a sensor for genotoxic stress [208] and *Lmna*^G609G/G609G^ mice (murine equivalent of *LMNA*^G608G/G608G^ mutation) exhibit activated NF-κB signaling through ATM-NEMO-mediated mechanisms [209].

#### 3.2.2. LEM Proteins in Cell Signaling

Several LEM proteins have been shown to recruit and regulate the transcriptional co-activators of the Wnt and the TGFβ signaling pathways: β-catenin and Smads, respectively:

- Emerin is a binding partner of β-catenin. Upon activation of Wnt signaling, β-catenin escapes proteasomal degradation and accumulates in the nucleus. Emerin binding to β-catenin inhibits its activity by facilitating nuclear export, thereby preventing accumulation in the nucleus in human fibroblasts [210].
- MAN1 binds to receptor-mediated Smads (rSmads), intracellular mediators of the TGF-β, and bone morphogenic protein (BMP) signaling. rSmads play an intimate role in cancer metastasis [211]. The C-terminus of MAN1 sequesters rSmads at the inner nuclear membrane, thereby preventing their ability to migrate to gene enhancer regions and activate transcription [212,213,214].

In brief, several elements of the NE provide an additional stage of regulation of signaling pathways that control proliferation and, in turn, potential points whose dysregulation may generate the unrestrained proliferation typical of tumor transformation.

## 4. Nuclear Envelope Regulation of the Genome

Tumor cells are characterized by massive changes in both the pattern of gene expression and in genome organization, the most critical of these being chromosome translocations and DNA damage/breaks. Since the link between chromosome translocations and tumorigenesis has been established in humans [215], checking karyotypes for translocations has become a standard diagnostic tool for many cancer types. Physically, chromosome translocations need DNA damage and repair systems to occur, but the course of chromosome translocations varies upon aspects of higher order chromosome structure in the nucleus. Particularly, this refers to chromosome positioning patterns. These patterns are preserved in a tissue-specific manner and might explain the preference for certain chromosomal translocations in certain tumor types. Indeed, in a particular tissue where the tumor arises, chromosomes adjacent one to another in the interphase nucleus will be involved in the chromosomal translocations distinctive of this tumor type [216].

### 4.1. Spatial Genome Organization Directed from the Nuclear Periphery

A direct physical contact between the NE and the chromatin was first suggested by microscopy observations [217,218]. Later biochemical experiments further supported that contact. In detail, chromatin components were retained and detected when extracting and purifying the NE using high ionic strength buffers [219].

More recently, new evidence in support of this view has come from specific chromatin epigenetic modifications. Heterochromatin is modified with histone H3 lysine 9 dimethylation and trimethylation (H3K9me2 and H3K9me3). In higher eukaryotes, antibodies against H3K9me2 reveal the distribution of heterochromatin close to the NE. Antibodies against H3K9me3, in contrast, stain internal heterochromatic domains in accordance with the presence of H3K9me3 in telomeres and centromeres [220]. These data agree with earlier biochemical and microscopic observations describing distinct subpopulations of HP1α at the NE in both mouse and human cells [221].

In addition, several chromatin-binding partners of NETs precisely colocalize with transcriptionally silenced chromatin [90,222,223]. Indeed, these partners are indispensable for heterochromatin distribution at the nuclear periphery in a specific pattern [50,224]. Mostly, the NET SUN proteins have been reported to mediate tethering to the NE of telomere and centromere in several systems [225,226,227,228,229].

### 4.2. Tethering of Chromosomes and Loci to the NE

NETs tissue-specific expression was previously described above. The specific collection of NETs in a cell type seems to be crucial in determining the position of chromosomes to the NE. Hence, when NETs preferentially expressed in muscle cells, fat, or liver are exogenously expressed in fibroblasts, chromosomes reposition to the NE. In liver cells, knock-down of liver-specific NETs 45 and 47 triggered the release of several chromosomes from the NE [230]. Strikingly, different NETs might affect the location of different subsets of chromosomes, raising the tempting possibility that NETs are the actual endogenous players behind a tissue-specific spatial genome organization [230].

### 4.3. Chromatin Lipid Fraction

The presence of phospholipids as a component of chromatin is well-documented and many enzymes, such as sphingomyelin-synthase (SMS) and sphingomyelinase (SMase), have been located in the INM [231]. Many different roles have been attributed to the intranuclear lipid fraction in relation to cell proliferation and differentiation.

Lipid microdomains, rich in Sphingomyelin (SM) and cholesterol (CHO), present in the INM are called nuclear lipid microdomains (NLMs). In the mammal liver, NLMs act as a resting place for active chromatin and transcription factors by regulating DNA [232] and RNA [8,233] synthesis.

SM is highly represented inside the nucleus and changes in its amount in different cell physiological states. These variations might reflect the nuclear presence of a SMase, which hydrolyzes SM to ceramide and phosphorylcholine (PPC), and a SMS, which synthesizes SM from ceramide and PPC derived from PC (reviewed by [231]). The prognostic value of SMS levels in certain types of human cancer is just beginning to be understood [234,235]. In the S-phase, the decondensation of chromatin is favored by a decrease of SM due to the increased activity of SMase. The decrease of SM favors the increase of the CHO free fraction. In turn, CHO enhances the activities of cyclin-dependent kinases needed for the entrance into the S-phase. In the contrast, inhibition of CHO synthesis induces cell cycle arrest in the S-phase with decreased expression of cdk2 and cdk4. At the end of the S-phase, an increase in SM is observed when SMS increases its activity whereas that of SMase decreases. Indeed, the restoration of the amount of SM marks the transition moment to the G2 phase (reviewed by [231]).

To recapitulate, NE actively participates in the distribution and organization of the genome within the nucleus, by means of NETs and specific membrane lipids. In the early stages of cancer, alterations in this distribution can result in chromosomal translocations and altered gene expression.

## 5. The Nuclear Envelope Link to Cell Migration and Metastasis and Its Use in Cancer Prognosis

The differences in cell morphology in human tumor cells were first reported in the mid-1800s [236,237]. Diagnostic features include differences in cell size and shape, number, and size of the nuclei, and loss of adherence to adjacent cells in biopsies [236,238]. In the mid-1900s, Papanicolau’s smear test was established as a routine technique for cervical cancer detection and is used today in a wide range of specimens, as outlined above [2,3,239]. Interestingly, in current computer-assisted diagnostic protocols, the diagnostic parameters employed are still largely morphological and nucleus centric, as they were 160 years ago. Focusing on the nucleus, the trained eye of the cytopathologist observes those features: Karyoplasmic ratio, nuclear roundness, NE smoothness, chromatin distribution, and the presence of NE invaginations and grooves (reviewed in [240]).

### 5.1. Nuclear Mechanics

Remarkably, while different cancers arise through different mechanisms and from different tissues, the above-mentioned nuclear abnormalities are mostly still common in all cancers, suggesting that nuclear structural alterations are functionally relevant in carcinogenesis. Mechanistically, the nucleoskeleton–cytoskeleton connection has profound effects on cell polarization, nuclear positioning, nuclear migration, and cell migration [241,242].

In addition to the implication of oncogenes and tumor-suppressor genes, the role of the physical constraints of tumor cells and their microenvironment has been explored within the last 15 years. Cancer cells show reduced stiffness, generate increased contractile forces, and are strongly influenced by their biomechanical environment (reviewed in [243]). Physical measurement can be used to distinguish nontumorigenic cells from cancer cells, and highly invasive from less invasive cells among those later [244].

The nucleus is both elastic and viscoelastic. Both behaviors are due to the nuclear lamina and the nuclear interior, respectively. Typically, the nucleus is ~2–10 times stiffer when compared to the surrounding cytoplasm. Hence, both the nuclear lamina and the nuclear interior govern the mechanical deformability of the nucleus. In reference to the lamins, cells modulate their levels according to the environmental constraints. Numerous studies on mice and cells grown on different supports show a prevalence of type A lamins in situations requiring stiffness, compared to a predominance of type B lamins when elasticity is required [245,246,247,248]. Moreover, lamins participate in the transmission of mechanical forces from the cytoskeleton, as they are integrated in a membrane–protein–chromatin network that allows their physical connection with Linkers of the Nucleoskeleton to the Cytoskeleton (LINC complexes) [249]. Additionally, chromatin contributes to nuclear stiffness and viscoelastic behavior (reviewed in [250]).

The structure and composition of the NE is especially relevant in cellular mechanics and function, affecting nuclear deformability and fragility and participating in mechanotransduction signaling (reviewed in [250]). Regarding the nuclear interior, it contains more than DNA and histones. The composition of this nucleoskeleton, its function, and its relevance in cancer remain a matter of debate, while a plethora of structural proteins are present on this compartment (actin, myosin, spectrin, γ-tubulin) (reviewed in [129,250]). Nucleo-cytoskeletal coupling has been reported to occur through LINC complexes, SUN domain proteins, nesprins and other KASH domain proteins, and the INM protein Samp1 [251,252]. These nucleo-cytoskeletal coupling elements are critical to ensure the force transmission between the nucleus and cytoskeleton and the modulation of the protrusions needed for cell migration (reviewed in [250]). Moreover, direct connections of the cytoskeleton with the nucleoskeleton through the NE might be an alternative means to the NPC for signal transduction between the cytoplasm and nucleus (reviewed in [240]). Using these elements, the stimulation of integrins on the surface of intact endothelial cells, for instance, results in both the reorientation of cytoskeletal filaments, the distortion of the nucleus, and the spatial redistribution of subnuclear structures [253]. In addition, changes in nuclear organization might affect gene expression of DNA stability (reviewed in [250]). Still, one open question is whether this mechanically induced change in nuclear structure and chromatin configuration can activate specifically mechanosensitive genes [254].

The relevance of nuclear mechanics in cancer comes from the fact that the nucleus is the largest and stiffest organelle of the cell, dominating the overall cellular mechanical response when cells are subjected to large deformations, for instance, when squeezing through narrow constrictions imposed by ECM fibers and other cells [241,242,255]. The irregular nuclear morphology of cancer cells often resembles that of abnormal nuclear shapes in cells with altered NE proteins, such as A- and B-type lamins and LBR (reviewed in [250]). Presumably, changes in nuclear architecture modify the rigidity of the nucleus, and this might increase nuclear deformability to benefit metastatic processes where cells need to pass through narrow interstitial spaces or small capillaries. Transient nuclear deformations, resulting in hourglass- and cigar-shape nuclei as well as nuclear protrusions have been observed during cancer cell migration in vivo [241].

### 5.2. Nuclear Positioning, Nuclear Envelope Rupture and Repair, and Cancer Cell Migration

Nuclear positioning in the cytoplasm is a highly regulated process that is dynamic in space and time and is required for multiple cellular and developmental processes. Extreme examples are skeletal muscle cells with a nuclei position at the periphery and epithelial tissues where nuclei are usually away from the apical membrane (reviewed in [256]). Nuclear positioning might additionally be involved in metastatic tumor cell migration, since cell polarization is required as a prior step for proper cell migration. In the course of cell polarization, in multiple migrating cell types, a centrosome and Golgi reorientation phenomenon occurs by which these organelles become positioned between the nucleus and the future leading edge. This reorientation seems to be achieved by a rearward movement of the nucleus away from the future leading edge, whereas the centrosome stays mostly static in the center of the cell [257]. Inhibition of nuclear movement impairs cell migration (reviewed in [256]).

Several molecular entities of the nucleo-cytoskeletal coupling have been found to be required for nuclear movement prior to cell migration: Nesprins, SUN, Samp1, and transmembrane actin- associated nuclear (TAN) lines, a novel NE structures involved in force transduction during nuclear movement (reviewed in [256]). The reshaping of the NE and the rearrangement of the NE-associated cytoskeleton help to prepare the cells for directional motion [258,259]. Furthermore, recent reports in mammals have connected the movement and positioning of organelles in the cytoplasm to γ-tubulin protein, e.g., in the positioning of Golgi apparatus and mitochondria [260,261]. In line with this, the Leu387Pro mutation in γ-tubulin was shown to influence nuclear positioning in yeast cells [262]. This may be attributed to the association of nuclear and cytosolic γ-strings with other components of the γ-tubulin meshwork, such as γ-tubules [263], which may provide a supporting scaffold dictating the positioning of the nuclear compartment.

A long list of translocations involving nucleoporins have been described in tumors (reviewed in [65,75]). Several of them alter the normal function of the NPC and contribute to pathogenicity. NUP214–ABL fusion protein needs to be targeted to the NPC for its transforming activity [264], and NUP98 fusion proteins affect nuclear export [265]. Furthermore, nucleoporins interact with important players in cell migration [143,266,267,268,269,270,271]. For example, NUP153 contributes to cell motility and migration interacting with A-type lamins and SUN1, while NUP358 interacts with kinesin 2 to locate APC to the cell cortex [267].

Concerning NE rupture and repair, in normal cells, NE breakdown and reassembly is limited to mitosis and is precisely regulated [105]. Missegregated chromosomes during mitotic exit can recruit their own NE to form micronuclei (MN), resulting in a NE susceptible to loss of integrity [272] and might be an objective biomarker for genomic instability in solid tumors. The irreversible NE rupture of MN can cause extensive DNA damage and promote tumorigenesis. MNs contain less NPC than the nucleus, are defective in nucleocytoplasmic transport, and replicate their DNA in an ineffective and asynchronous manner to their primary nucleus [272,273]. Furthermore, in many cancer cells, transient rupture and resealing of the NE is a common event during interphase. As a result, the nucleus and cytoplasm temporarily exchange material between them, even occasionally entrapping cytoplasmic organelles inside the nucleus. NE rupture has been further linked to the appearance of micronuclei, to the mislocalization of nucleoplasmic and cytoplasmic proteins, and to the exit of chromatin portions from the nuclear interior [274].

### 5.3. Nuclear Envelope Invaginations and Cancer

The NE is mainly a smooth surface, but it also presents invaginations. Those invaginations can stretch deep into the nucleoplasm, eventually crossing the nucleus entirely. High-resolution microscopy observations have revealed that aberrations in nuclear shape and size used for cancer diagnosis are probably due to large-scale invaginations of the NE that are able to traverse most or even all the nucleus (reviewed in [275]). These invaginations have been named the nucleoplasmic reticulum (NR) for their morphological similarity to the ER. NR invaginations are called type I when the INM alone is involved, while NR invaginations are called type II when both the INM and ONM invaginate into the nucleoplasm. Type II invaginations may enclose microtubules, microfilaments, and even mitochondria in their cytoplasmic core [276,277]. Interestingly, a potential implication of γ-tubulin tubules in nuclear invagination may be further investigated, since a recent report showed that γ-tubules could also pass through the center of the nucleus [261]. The NR is observed in nuclei from various normal and abnormal tissues [278], as well as in cells grown in 2D and 3D cultures, including many tumor cell types, such as brain, breast, kidney, bladder, prostate, and ovary [279,280]. Appearance of the NR may occur both after mitosis, during NE reassembly, and without mitosis, i.e., de novo in interphase cell nuclei. Then, NR is commonly maintained throughout interphase and, in specific cell types, it can show heritable patterns [281,282,283]. The actual mechanisms by which this new nuclear structural component, the NR, affects cell function in normal and cancer cells are still under investigation. However, the diagnostic and prognostic significance of irregularities and invaginations of the NE in cancer cells are indisputable.

## 6. Conclusions

The specific traits of nuclear cell morphology in tumor cells have been used in the last 150 years to evaluate the diagnosis and prognosis of human cancer. Although the NM, attached NETs, and other proteins have been found to be implicated in this morphology (e.g., type A/C and type B lamins, nucleoporins, γ-tubulin), further research is needed to precisely define the molecular entities behind the alterations in the number, size, and shape of nuclei in tumor cells.

## Abbreviations

APC	Adenomatous polyposis coli (APC)
BAF	Barrier-to- autointegration-factor
BMP	Bone morphogenic protein
CCT	Chaperonin containing TCP-1
CDK	Cyclin-dependent kinase
CHO	Cholesterol
ECM	Extracellular matrix
EDMD	Emery–Dreifuss muscular dystrophy
ER	Endoplasmic reticulum
ERK1/2	Extracellular signal-regulated kinase 1/2
ESCRT	Endosomal sorting complexes required for transport
FG	Phenylalanine and glycine
HGPS	Hutchinson–Gilford progeria syndrome
HP1	Heterochromatin protein 1
INM	Inner nuclear membrane
JNK	c-Jun NH2-terminal kinase
LADs	Lamina-associated domains
LBR	Lamin B receptor
MAPK	Mitogen Activated Protein Kinase
MN	Micronuclei
NE	Nuclear envelope
NETs	Nuclear envelope transmembrane proteins
NLMs	Nuclear lipid microdomains
NPCs	Nuclear pore complexes
NR	Nucleoplasmic reticulum
OIS	Oncogene-induced senescence
ONM	Outer nuclear membrane
PPC	Phosphorylcholine
RB	Retinoblastoma
SAHFs	Senescence-associated heterochromatin foci
SENP1	Sentrin-specific protease 1
SM	Sphingomyelin
SMS	Sphingomyelin-synthase
TAN lines	Transmembrane actin- associated nuclear lines
TGFβ	Transforming growth factor-β
